# Influence of climate change and postdelisting management on long‐term population viability of the conservation‐reliant Kirtland's Warbler

**DOI:** 10.1002/ece3.5547

**Published:** 2019-08-24

**Authors:** Donald J. Brown, Deahn M. Donner, Christine A. Ribic, Carol I. Bocetti

**Affiliations:** ^1^ School of Natural Resources West Virginia University Morgantown WV USA; ^2^ Northern Research Station U.S.D.A. Forest Service Parsons WV USA; ^3^ Northern Research Station U.S.D.A. Forest Service Rhinelander WI USA; ^4^ U.S. Geological Survey Wisconsin Cooperative Wildlife Research Unit Department of Forest and Wildlife Ecology University of Wisconsin‐Madison Madison WI USA; ^5^ Department of Biological and Environmental Sciences California University of Pennsylvania California PA USA

**Keywords:** Bahamas, bird, jack pine, Michigan, migratory, *Setophaga kirtlandii*, simulation model, STELLA

## Abstract

Rapid global climate change is resulting in novel abiotic and biotic conditions and interactions. Identifying management strategies that maximize probability of long‐term persistence requires an understanding of the vulnerability of species to environmental changes. We sought to quantify the vulnerability of Kirtland's Warbler (*Setophaga kirtlandii*), a rare Neotropical migratory songbird that breeds almost exclusively in the Lower Peninsula of Michigan and winters in the Bahamian Archipelago, to projected environmental changes on the breeding and wintering grounds. We developed a population‐level simulation model that incorporates the influence of annual environmental conditions on the breeding and wintering grounds, and parameterized the model using empirical relationships. We simulated independent and additive effects of reduced breeding grounds habitat quantity and quality, and wintering grounds habitat quality, on population viability. Our results indicated the Kirtland's Warbler population is stable under current environmental and management conditions. Reduced breeding grounds habitat quantity resulted in reductions of the stable population size, but did not cause extinction under the scenarios we examined. In contrast, projected large reductions in wintering grounds precipitation caused the population to decline, with risk of extinction magnified when breeding habitat quantity or quality also decreased. Our study indicates that probability of long‐term persistence for Kirtland's Warbler will depend on climate change impacts to wintering grounds habitat quality and contributes to the growing literature documenting the importance of considering the full annual cycle for understanding population dynamics of migratory species.

## Graphical abstract

We sought to quantify the vulnerability of the rare Neotropical migratory songbird Kirtland's Warbler to projected environmental changes on the breeding and wintering grounds. We simulated independent and additive effects of reduced breeding grounds habitat quantity and quality, and wintering grounds habitat quality, on population viability. Projected large reductions in wintering grounds precipitation caused the population to decline, with risk of extinction magnified when breeding habitat quantity or quality also decreased.

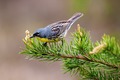

## 1 INTRODUCTION

Rapid climate changes are occurring at the global scale (IPCC, 2013). These changes have the potential to dramatically alter ecological communities through the introduction of spatially and temporally novel abiotic conditions and biotic interactions (Blois, Zarnetske, Fitzpatrick, & Finnegan, 2013; Gibson‐Reinemer, Sheldon, & Rahel, 2015). Species that undertake annual long‐distance migrations, such as Neotropical migratory songbirds, may be particularly vulnerable to rapid climate change because they rely on environmental conditions in multiple spatially discrete areas and the corridors between them (Carey, 2009). Further, environmental conditions in one portion of the annual cycle can impact individual fitness directly or have delayed effects that carry over to other portions of the annual cycle (Paxton & Moore, 2015; Rockwell, Bocetti, & Marra, 2012).

The Upper Midwest, USA, is a major breeding region for Neotropical migratory songbirds, particularly the states of Michigan, Wisconsin, and Minnesota (Niemi et al., 2016; Thompson, Lewis, Green, & Ewert, 1993). Projection models indicate that tree communities in the Upper Midwest will change over the next century in response to climate change (Handler et al., 2014; Iverson, Prasad, Matthews, & Peters, 2008). Bird species distributions will likely expand, contract, or shift in response to climate and forest composition changes (Cumming et al., 2014; Schulte, Pidgeon, & Mladenoff, 2005), and regional abundances could decrease for many species (Matthews, Iverson, Prasad, & Peters, 2011).

On the wintering grounds, availability of food resources can regulate population dynamics by influencing annual survival and fecundity (Holmes, 2007; Norris, Marra, Kyser, Sherry, & Ratcliffe, 2004). In the neotropics, food resource availability has been shown to influence body condition of migratory birds and to be positively correlated with precipitation (Studds & Marra, 2007; Wunderle, Lebow, White, Currie, & Ewert, 2014). As the climate changes, spatial and temporal weather patterns in the neotropics are changing, with seasonal drying trends in some regions (Karmalkar et al., 2013; Wolcott, Donner, Brown, & Ribic, 2018). Decreases in precipitation during the spring premigration period could have particularly strong negative impacts on migratory bird populations (Rockwell et al., 2017; Strong & Sherry, 2000).

**Figure 1:**
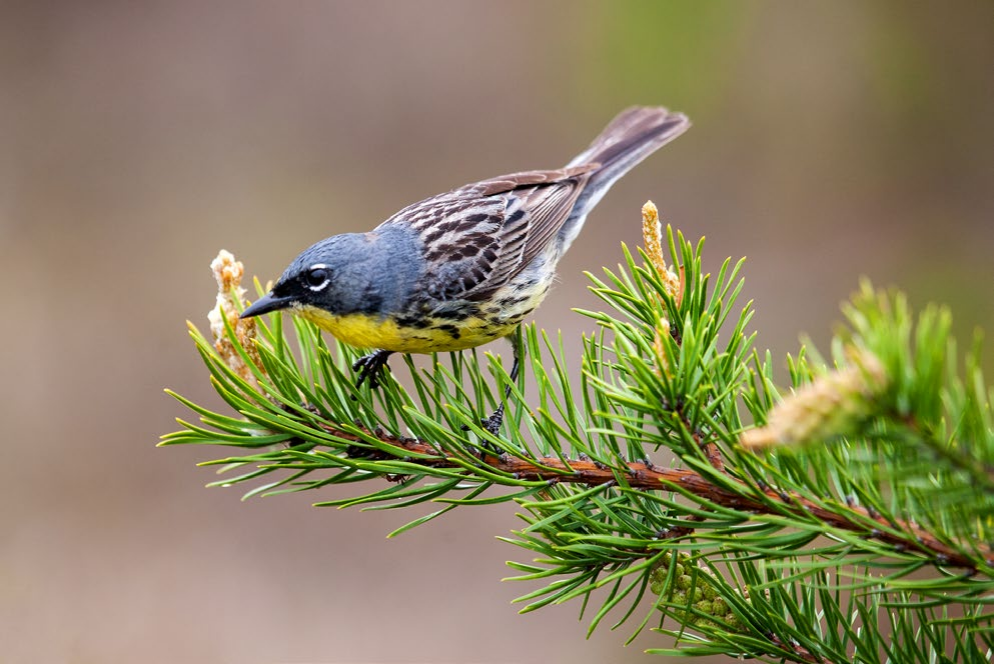
An adult male Kirtland's Warbler (*Setophaga kirtlandii*) perched on a jack pine (*Pinus banksiana*) branch on its breeding grounds in the northern Lower Peninsula of Michigan. Photograph used with permission from Nathan W. Cooper

One Neotropical migratory bird that is potentially highly vulnerable to climate change is the Kirtland's Warbler (*Setophaga kirtlandii*; Figure 1), a species that breeds almost exclusively in the northern Lower Peninsula (LP) of Michigan, and winters in the Bahamian archipelago (Cooper, Hallworth, & Marra, 2017; Probst, 1986). Kirtland's Warbler is considered the rarest songbird in North America (Wilson, Marra, & Fleischer, 2012) and has been listed as federally endangered since 1970 (Bureau of Sports Fisheries & Wildlife, 1970). Due to aggressive and collaborative management actions, coupled with the pivotal >24,000‐ha Mack Lake wildfire, the species has recovered from ca. 200 breeding males in 1971 to over 2,000 breeding males today (Bocetti, Goble, & Scott, 2012) and is currently proposed to be delisted under the Endangered Species Act.

Kirtland's Warbler is a habitat specialist on the breeding grounds, largely restricted to glacial outwash plains containing well‐drained sandy soils and dominated by large stands of young, dense jack pine (*Pinus banksiana*). A recent forest vulnerability assessment concluded that the vulnerability of jack pine to climate change is high–moderate in Michigan, based on projections from several different models (i.e., Climate Change Tree Atlas, LANDIS‐II, PnET‐CN; Handler et al., 2014). A subsequent study using Random Forest models projected that climatic suitability will decrease for jack pine in the Upper Midwest as precipitation and temperature increase (Donner, Brown, Ribic, Nelson, & Greco, 2018).

On their wintering grounds, Kirtland's Warblers subsist on fruiting shrubs and arthropods (Wunderle, Currie, & Ewert, 2007; Wunderle et al., 2010), with abundances of these food resources positively correlated with precipitation (Wunderle et al., 2014). Empirical studies found that premigration (i.e., March) precipitation was positively correlated with subsequent fledgling production and adult annual survival (Rockwell et al., 2012, 2017). Wolcott et al. (2018) summarized end‐of‐century climate change projections for the Bahamian Archipelago, which indicated that March temperature will increase and March precipitation could decrease in the central islands that serve as the core wintering grounds habitat for Kirtland's Warblers (Cooper et al., 2017).

In addition to potential climate change impacts, future changes in management of Kirtland's Warbler breeding grounds habitat could impact long‐term population viability. The recovery of the species can be attributed to two major management initiatives: increasing recruitment through removal of Brown‐headed Cowbirds (*Molothrus ater*), an obligate nest parasite, and increasing carrying capacity through creation of breeding habitat (DeCapita, 2000; Donner, Ribic, & Probst, 2008). Funding has been secured to support postdelisting continuation of the Brown‐headed Cowbird removal program (U.S. Fish & Wildlife Service, 2018). Breeding habitat was historically generated naturally through large wildfires with ca. 60 year return intervals (Cleland et al., 2004), but due to broad‐scale fire suppression, an extensive network of plantations is currently required to maintain large stands of young, dense jack pine (Bocetti, 1994; Donner, Ribic, & Probst, 2009).

As part of the postdelisting management strategy, there is interest in modifying the plantation program to reduce planting costs and increase timber value. The Kirtland's Warbler Breeding Range Conservation Plan proposes using experimental planting techniques for up to 25% of future created habitat (e.g., reduced tree densities, changes in habitat configuration, mixed species plantations; Michigan Department of Natural Resources, U.S. Fish, and Wildlife Service, & U. S. Forest Service, 2014). Because current planting prescriptions are designed to maximize habitat quality for the Kirtland's Warbler, modifications will likely negatively impact both density of males and pairing success, thus affecting carrying capacity and productivity of the warbler (Bocetti, 1994; Probst & Hayes, 1987).

In a previous study, we projected the influence of potential breeding grounds management changes on long‐term population viability of the Kirtland's Warbler, while also accounting for the influence of dynamic wintering grounds habitat quality based on contemporary climate conditions (Brown et al., 2017). In this study, we build from that initial work by projecting independent and additive effects of climate and management changes on long‐term population viability of the Kirtland's Warbler. To achieve this objective, we developed a population‐level simulation model that incorporates the influence of annual environmental conditions on the breeding and wintering grounds, and investigated effects of reduced breeding grounds habitat quantity and quality, and wintering grounds habitat quality, on population viability. We did not consider effects of changes in wintering grounds habitat quantity because carrying capacity of Kirtland's Warblers is strongly associated with food availability, which varies annually and seasonally due to weather variability (Wunderle et al., 2014).

## 2 METHODS

### 2.1 Study area

**Figure 2:**
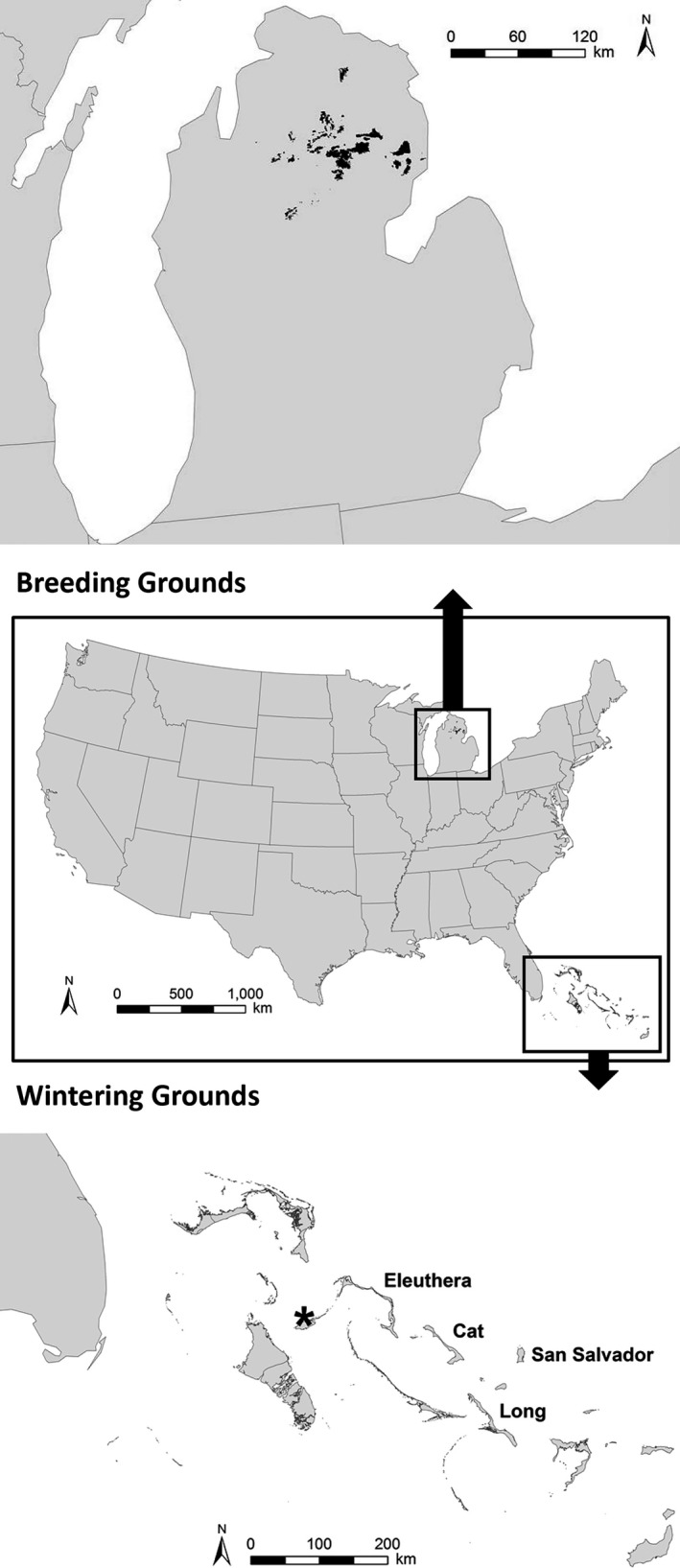
Focal Kirtland's Warbler (*Setophaga kirtlandii*) breeding grounds and wintering grounds habitat included in this study assessing vulnerability of the species to potential future changes in environmental and management conditions. The breeding grounds included designated essential breeding habitat on federal and state lands in the Lower Peninsula (LP) of Michigan, USA, which serves as the core breeding habitat for the species and contains >95% of all breeding individuals. The wintering grounds included the four Bahamian islands with the highest abundances of Kirtland's Warblers based on current monitoring data (i.e., Cat, Eleuthera, Long, and San Salvador). The * delineates the location of the Nassau National Oceanic and Atmospheric Administration (NOAA) weather station

Our breeding grounds study area consisted of designated essential Kirtland's Warbler breeding habitat on federal and state lands in the LP of Michigan, USA (Kirtland's Warbler Management Areas [KWMAs]; Byelich et al., 1985; Figure 2). This region is the core breeding habitat for the species and contains >95% of all breeding individuals (U.S. Fish & Wildlife Service, 2012). In addition, nearly all demographic data for the Kirtland's Warbler has come from this region. Additional currently occupied breeding areas (i.e., additional private lands in the LP, Upper Peninsula of Michigan (Probst, Donner, Bocetti, & Sjogren, 2003), Wisconsin (Anich, Trick, Grveles, & Goyette, 2011), and Ontario (Richard, 2014)) were not included because the majority of these lands are not managed specifically for Kirtland's Warbler and long‐term habitat availability is unpredictable. Thus, our estimated available breeding habitat was slightly conservative with respect to range‐wide breeding patch occupancy, but realistic given expected consistent long‐term habitat availability. Our wintering grounds study area included Cat, Eleuthera, Long, and San Salvador islands, the four Bahamian islands with the highest densities of Kirtland's Warblers based on current monitoring data (Cooper, Ewert, Wunderle, Helmer, & Marra, 2019; Figure 2).

### 2.2 Modeling approach

We developed a model that linked population demographics with temporally dynamic environmental conditions on the breeding and wintering grounds, using the program STELLA Professional (version 1.7.1; isee systems). STELLA is a dynamic systems modeling program that is capable of handling complex model structures, such as subtime steps (e.g., seasons within years), variable time step lengths, and feedback loops (e.g., Rodenhouse, 1992). Thus, it is a natural fit for modeling full annual cycle dynamics, as the annual cycle can be broken into multiple discrete stages, each with their own potentially dynamic environmental conditions and influence on population vital rates.

**Figure 3:**
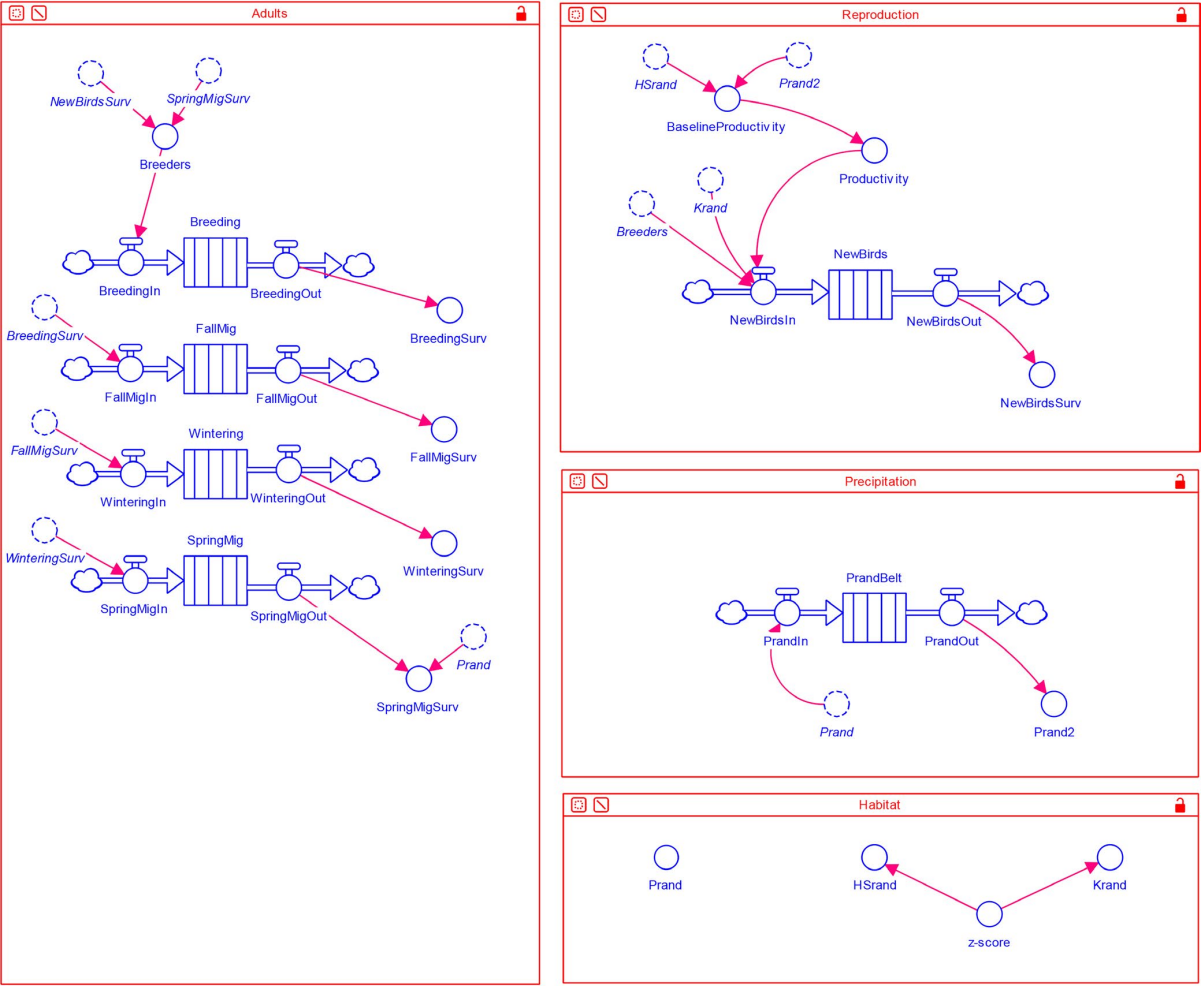
Kirtland's Warbler (*Setophaga kirtlandii*) population simulation model developed using the program STELLA Professional (version 1.7.1). The model contains stocks (rectangles) that hold birds during discrete time steps, flows (clouds and regulators connected by arrows) that move birds into and out of stocks (potentially using an equation, such as NewBirdsIn), converters (circles) that contain values or equations and influence flows, and connectors (arrows) that link the model components. Converters with broken lines indicate “ghosts,” where the original converter is located elsewhere in the model. The “Adults” submodel tracks movement of adults through the annual cycle. Adults and juveniles that survive through the nonbreeding period are added as potential breeders each year, and adult annual survival is influenced by precipitation on the wintering grounds. The “Reproduction” submodel simulates the new breeders produced each year. Annual productivity is influenced by precipitation on the wintering grounds, breeding grounds habitat suitability, breeding grounds carrying capacity, and number of potential breeders. The “Precipitation” submodel is used for tracking precipitation in year X and X−1. The “Habitat” submodel contains statistical distributions to draw annual values for wintering grounds precipitation (Prand), breeding grounds habitat suitability (HSrand), and breeding grounds carrying capacity (Krand). Each year, *z*‐score values are drawn and used to obtain the corresponding HSrand and Krand values, whereas Prand values are independently drawn

In our model, the Kirtland's Warbler population moved through four subtime steps annually: breeding grounds, fall migration, wintering grounds, and spring migration (Figure 3). Environmental conditions on the breeding grounds influenced productivity, and environmental conditions on the wintering grounds influenced survival and productivity. For Kirtland's Warbler, currently there are no quantitative data linking dynamic environmental conditions during the migratory periods to population vital rates, but the model can accommodate this information in the future. Most of the data available for model parameterization were based on male observations, and thus, we used a single‐sex model based on male empirical data for this study.

#### 2.2.1 Annual survival

Adult male annual survival was modeled as a function of wintering grounds habitat quality, using model estimates from an empirical study that found total March precipitation recorded at the Nassau National Oceanic and Atmospheric Administration (NOAA) weather station in the Bahamas in breeding year X was a strong predictor of survival probability to breeding year X + 1 (Rockwell et al., 2017). Specifically, we used the estimated parameters for a model that included mean annual survival as a function of March precipitation. For this model, baseline adult male annual survival was 0.4544 and increased by 0.0395 with every 1 cm increase in precipitation. We bounded maximum annual survival by the highest empirical estimate of 0.75 (Probst, 1986). Currently, there are no quantitative data linking wintering grounds habitat quality to hatch‐year survival, and thus, we used a constant value of 0.35, representing estimated average hatch‐year survival under current environmental conditions (Rockwell et al., 2017).

#### 2.2.2 Annual productivity

Annual per capita fledgling production was modeled as a function of wintering and breeding grounds habitat quality (Appendix 1). Rockwell et al. (2012) found total March precipitation recorded at the Nassau NOAA weather station was a strong predictor of subsequent male fledgling production. Habitat quality on the breeding grounds influences productivity by affecting pairing success (Probst, 1986; Rockwell, 2013). To allow both wintering and breeding grounds habitat quality to influence production of new males, our equation included two predictive components. The first component estimated baseline productivity based on mean annual breeding grounds habitat quality (0.678 + 0.1334 [HQ]; Appendix 1). The second component estimated productivity above the baseline with every 1 cm increase in precipitation (0.168 [P]). We note this is the same approach used in Brown et al. (2017), but the equations differ because here we modeled hatch‐year survival separately, rather than accounting for survival in a prebreeding census matrix model. We bounded maximum fledgling production by the highest empirical estimate of 2.19 male fledglings per male (Shake & Mattsson, 1975).

#### 2.2.3 Carrying capacity

Annual carrying capacity on the breeding grounds is dependent on both quantity and quality of breeding habitat. Habitat quality, a function of stand density and age, influences density of Kirtland's Warblers (Bocetti, 1994; Probst, 1986). The Kirtland's Warbler Conservation Team defined 14,593 ha as the target for annual breeding habitat availability (U.S. Fish & Wildlife Service, 2018), and we used this as our baseline annual breeding habitat quantity. To obtain annual breeding grounds carrying capacity, we multiplied quantity of breeding habitat by mean density, which we simulated using a Gaussian distribution based on empirical data from 2004 to 2017 (Appendix 1). Thus, the model accounted for annual variability in mean quality of habitat, reflected in changes in mean density. We assumed ceiling‐type density dependence, which allows populations to grow exponentially until they reach carrying capacity (Akçakaya et al., 2004). When the population exceeded the carrying capacity, reproduction was restricted to the carrying capacity, with the remaining individuals allowed to survive, but not reproduce. Thus, the population could exceed the model carrying capacity, but at the cost of reduced per capita productivity.

#### 2.2.4 Stochasticity

We incorporated environmental stochasticity in breeding grounds habitat quality and wintering grounds precipitation. In our model, annual productivity and carrying capacity are both influenced by habitat quality, but use different Gaussian distributions (i.e., productivity uses a habitat quality index and carrying capacity uses mean density of Kirtland's Warblers). To align annual habitat quality for the two parameters, we specified the model to draw a habitat quality value from a *z*‐distribution (mean = 0, *SD* = 1) and then transformed the *z*‐score to create the corresponding value for each distribution: Value = mean + (*z*‐score × *SD*).

For wintering grounds precipitation, we obtained total March precipitation recorded at the Nassau NOAA weather station from 1994 to 2013. We removed the upper 10% of observation years from the data set because they represented low‐frequency extreme precipitation years (e.g., 21.02 cm recorded in 2001). We acknowledge that extreme precipitation years likely influence population vital rates, but currently there are no data to infer the magnitude, or even direction, of these relationships. For the projection models, annual wintering grounds precipitation was drawn from a Gaussian distribution parameterized using the empirical mean and standard deviation, with the lower tail bounded at 0. In addition to environmental stochasticity, we incorporated demographic stochasticity in adult and hatch‐year annual survivorship by drawing values from a binomial distribution that was parameterized using initial abundance and model‐predicted (adult) or specified (hatch‐year) mean annual survival (Akçakaya, 1991).

#### 2.2.5 Model validation

**Figure 4:**
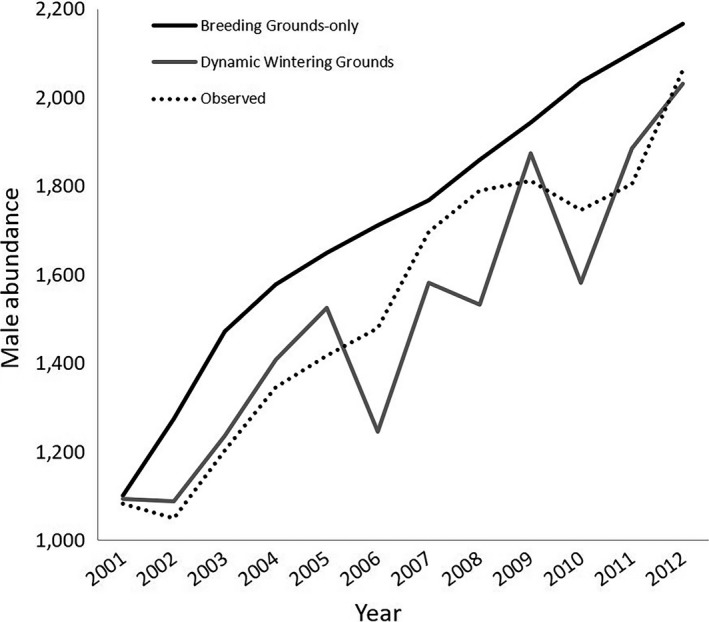
Deterministic simulations of abundance of male Kirtland's Warblers (*Setophaga kirtlandii*) from 2001–2012 using a model that only incorporated dynamic environmental conditions on the breeding grounds (Breeding Grounds‐only; solid black line), and a model that incorporated dynamic environmental conditions on the breeding grounds and temporal variation in wintering grounds habitat quality (Dynamic Wintering Grounds; solid gray line). For the Dynamic Wintering Grounds model, we used observed total March precipitation from 2004–2012, and mean precipitation from the projection model (i.e., 3.42 cm) for 2001–2003 due to these being extreme precipitation years, for which the relationships between precipitation and population vital rates are unknown. Observed abundances represent the total counts in Michigan from annual Kirtland's Warbler breeding male censuses (black dotted line)

We tested if incorporating temporal variation in wintering grounds habitat quality improved model performance compared to only considering dynamic environmental conditions on the breeding grounds. For this comparison, we created a breeding grounds‐only model, with annual survival and fledgling production representing the mean estimates from Rockwell et al. (2012) and Rockwell et al. (2017). We then deterministically simulated abundances from 2001 to 2012 using observed environmental data and compared the output of each model to observed abundances from the annual Kirtland's Warbler breeding male census (U.S. Fish & Wildlife Service, 2015). Incorporating dynamic wintering grounds habitat quality resulted in a more accurate reconstruction of historical abundances (sum of squared deviations from observed abundance = 496,237 and 189,940 for the breeding grounds‐only and dynamic wintering grounds model, respectively; Figure 4).

#### 2.2.6 Future projections

We used our simulation model to project Kirtland's Warbler population dynamics based on contemporary and potential future environmental and management conditions. We tested independent and additive effects of changes in wintering grounds habitat quality and breeding grounds habitat quantity and quality (Table 1). To model influences of future environmental changes, we modified contemporary environmental distributions by multiplying their annual values by a proportional change value, thus retaining environmental stochasticity while allowing for directional changes. We did not include a gradual change in environmental values from contemporary to future conditions.

**Table 1:** Simulated independent and additive effects of changes in wintering grounds habitat quality (i.e., reduced March precipitation; WQL), breeding grounds habitat quantity (reduced jack pine suitability due to climate [BQN‐C] or experimental plantations [BQN‐M]), and breeding grounds habitat quality (experimental plantations; BQL) on Kirtland's Warbler population dynamics

Model	Parameter changes			
	Wintering habitat quality‐ Climate (WQL)	Breeding habitat quantity‐ Climate (BQN‐C)	Breeding habitat quantity‐ Experimental plantations (BQN‐M)	Breeding habitat quality‐ Experimental plantations (BQL)
Contemporary				
WQL	X			
BQN‐C		X		
BQN‐M			X	
BQL				X
WQL + BQN‐C	X	X		
WQL + BQN‐M	X		X	
WQL + BQL	X			X
BQN‐C + BQL		X		X
WQL + BQN‐C + BQL	X	X		X

Note

Each scenario involving reduced wintering grounds habitat quality was run with three levels of precipitation change: median change: −0.5%, lower 25th percentile: −32.0%, lower 10th percentile: −55.1%, based on an ensemble of 40 general circulation models and Representative Concentration Pathway 8.5. The contemporary model assumes environmental conditions remain the same on the wintering and breeding grounds, and thus serves as a null model for comparing impacts of climate and management changes.

To estimate potential climate change impacts on wintering grounds habitat quality, we projected proportional changes in average March precipitation in 2100 relative to baseline precipitation for the focal Bahamian islands (Appendix 1). We used an ensemble of 40 GCMs, with mid‐equilibrium climate sensitivity levels and Representative Concentration Pathway (RCP) 8.5. We used the median (−0.055), lower 25th percentile (−0.331), and lower 10th percentile (−0.558) of projected end‐of‐century changes in March precipitation based on the GCM ensemble.

To estimate potential climate change impacts on breeding grounds habitat quantity, we projected end‐of‐century suitable habitat for jack pine occurrence in the LP of Michigan based on median end‐of‐century climate estimates from 31 GCMs and RCP 8.5 (Donner et al., 2018). We then determined the proportion of contemporary Kirtland's Warbler habitat (2004–2013; Brown et al., 2017) that was located in areas that are projected to become climatically unsuitable for jack pine. This resulted in a mean reduction in breeding habitat of 48.7 ± 3.2%.

To estimate potential management change impacts on breeding grounds habitat quantity, we used a reduction in breeding habitat quantity of 25% (Michigan Department of Natural Resources, U.S. Fish, and Wildlife Service, & U. S. Forest Service, 2014). This represents a worst‐case management scenario, where experimental plantations do not provide habitat for Kirtland's Warblers. To estimate potential management change impacts on breeding grounds habitat quality, we replaced 25% of high suitability habitat with low suitability habitat, thus reducing mean habitat suitability. This scenario assumes that experimental plantations continue to provide habitat for Kirtland's Warblers, but habitat quality is reduced.

For each model scenario, we projected the population for 100 years and completed 1,000 replications. To estimate long‐term population growth, we computed annual growth rates over the 100‐year simulation based on mean abundance at each time step (*λ* = *N_t_*_+1_/*N_t_*). We then calculated the geometric mean and 95% confidence interval for *λ* (based on a *t*‐distribution; Stevens, 2009). To estimate the risk of extinction for model scenarios, we computed the proportion of replicates where abundance declined to 0 over the 100 years simulation period. We also computed the proportion of replications where abundance fell below 200 males, which represents the critical low abundance observed in the early 1970s, and 1,000 males, which represents the species recovery plan goal (Byelich et al., 1985), and remains the perpetual goal for minimum number of breeding pairs (U.S. Fish & Wildlife Service, 2018).

## 3 RESULTS

**Figure 5:**
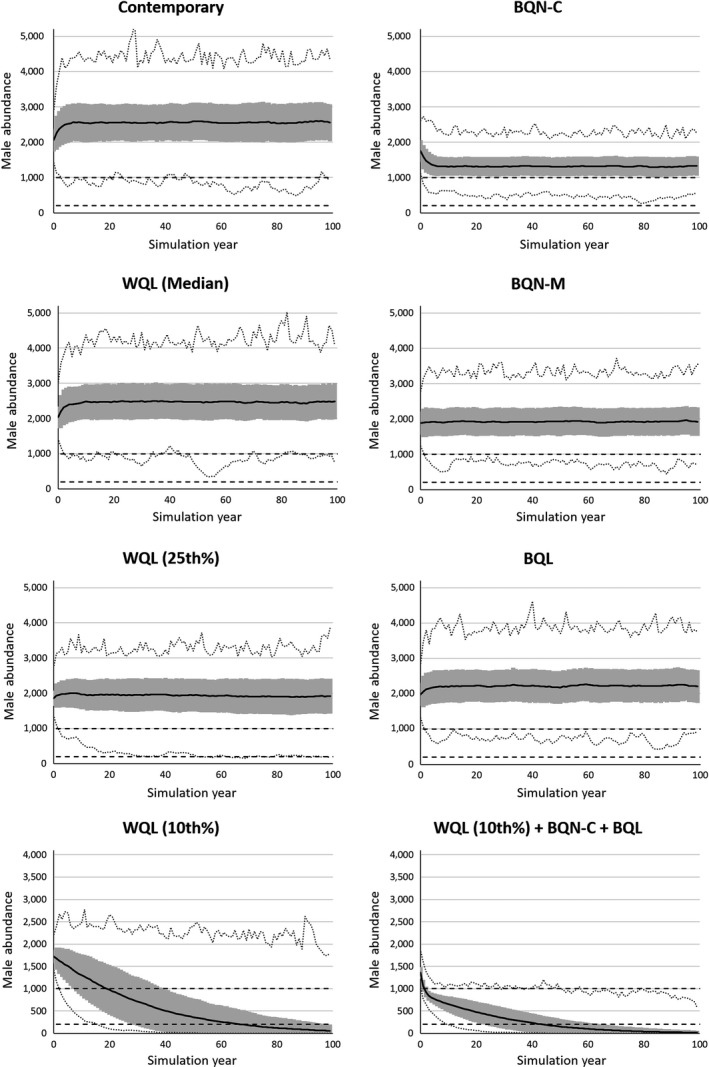
Impacts of simulated effects of environmental and management changes on abundance of the Kirtland's Warbler (*Setophaga kirtlandii*) over 100 years. Models include reduced wintering grounds habitat quality (WQL; median, 25th percentile, and 10th percentile of climate models projecting end‐of‐century changes in March precipitation under Representative Concentration Pathway 8.5), reduced breeding grounds habitat quantity due to climate (BQN‐C) and management (BQN‐M) changes, and reduced breeding grounds habitat quality due to management changes (BQL). The contemporary model reflects current environmental and management conditions on the wintering and breeding grounds. The WQL (10th%) + BQN‐C + BQL model represents that most extreme environmental change of all modeled scenarios. The dark solid line shows the mean number of males per year from 1,000 independent simulations. The gray lines show the standard deviation each year, and the dotted lines show the maximum and minimum number of males each year across all simulations. The dark horizontal dashed lines represent the management target of 1,000 males and critical threshold of 200 males

Under contemporary environmental and management conditions, the population was stable over the 100‐year simulation period (Table 2), with a mean annual abundance of ca. 2,500 males (Figure 5). Under contemporary environmental and management conditions, 4.9% of simulations resulted in a population that fell below the management target of 1,000 males, but no simulations fell below the critical threshold of 200 males.

**Table 2:** Impacts of simulated independent and additive effects of environmental and management changes on population growth rate (*λ*) and abundance thresholds for the Kirtland's Warbler (*Setophaga kirtlandii*)

Model	*λ*	95% CI	*N* < 1,000 (%)	*N* < 200 (%)	*N* = 0 (%)
Contemporary	1.0022	0.9998–1.0045	4.9	0	0
WQL (Median)	1.0020	0.9999–1.0042	5.2	0	0
WQL (25th%)	1.0003	0.9990–1.0016	39.2	0.3	0
WQL (10th%)	0.9661	0.9647–0.9676	99.7	95.0	5.5
BQN‐C	0.9972	0.9944–1.0000	99.6	0	0
BQN‐M	1.0002	0.9994–1.0010	21.7	0	0
BQL	1.0011	0.9997–1.0025	9.1	0	0
WQL (Median) + BQN‐C	0.9970	0.9941–0.9999	99.7	0	0
WQL (25th%) + BQN‐C	0.9954	0.9915–0.9994	100	2.6	0
WQL (10th%) + BQN‐C	0.9622	0.9584–0.9660	100	98.1	9.9
WQL (Median) + BQN‐M	0.9998	0.9990–1.0007	26.3	0	0
WQL (25th%) + BQN‐M	0.9983	0.9970–0.9997	77.8	0.7	0
WQL (10th%) + BQN‐M	0.9666	0.9649–0.9682	99.9	95.2	6.2
WQL (Median) + BQL	1.0011	0.9997–1.0024	13.0	0	0
WQL (25th%) + BQL	0.9992	0.9982–1.0001	58.1	0.8	0
WQL (10th%) + BQL	0.9631	0.9619–0.9644	99.7	97.3	10.6
WQL (Median) + BQN‐C + BQL	0.9958	0.9919–0.9997	100	0.1	0
WQL (25th%) + BQN‐C + BQL	0.9942	0.9893–0.9991	100	5.1	0
WQL (10th%) + BQN‐C + BQL	0.9569	0.9524–0.9615	100	99.1	17.7
BQN‐C + BQL	0.9959	0.9921–0.9997	100	0	0

Note

Models include reduced wintering grounds habitat quality (WQL; median, 25th percentile, and 10th percentile of climate models projecting end‐of‐century changes in March precipitation based on an ensemble of 40 general circulation models and Representative Concentration Pathway 8.5), reduced breeding grounds habitat quantity due to climate (BQN‐C) and management (BQN‐M) changes, and reduced breeding grounds habitat quality due to management changes (BQL). The contemporary model reflects current environmental and management conditions on the wintering and breeding grounds.

For independent effects of projected end‐of‐century changes in March precipitation, the population remained stable with the median and lower 25th percentile of projected changes, and declined with the 10th percentile of projected changes (Table 2, Figure 5). For the lower 25th percentile, the mean final abundance was 1,920 males, with 39.2% of simulations falling below the management target of 1,000 males (Table 2, Figure 5). For the 10th percentile projected change, the mean final abundance was 57 males, with 95.0% of simulations falling below the critical threshold of 200 males (Table 2, Figure 5).

When quantity of breeding grounds habitat was reduced due to climate change, the population initially declined, but stabilized at ca. 1,300 males (Figure 5). Similarly, when quantity of breeding grounds habitat was reduced due to management changes, the population initially declined, but stabilized at ca. 1,900 males (Figure 5). While loss of breeding grounds habitat lowered the carrying capacity, additional factors were necessary for the population to decline below the critical threshold of 200 males. The population remained stable at ca. 2,200 males when breeding grounds habitat quality was reduced due to management changes (Table 2, Figure 5).

For additive effects, only three scenarios resulted in potentially stable populations based on 95% confidence intervals (Table 2). The additive scenario where the population was most likely to remain above 1,000 males included a median projected change in precipitation and reduction in breeding grounds habitat quality due to management changes, which had a mean final abundance of 2,142 males, and 13.0% of simulations fell below 1,000 males. Reducing precipitation to the lower 25th percentile of projected changes also resulted in a potentially stable population, but with 58.1% of simulations falling below 1,000 males and 0.8% falling below 200 males. The other scenario resulting in a potentially stable population included a median projected change in precipitation and reduction in breeding grounds habitat quantity due to management changes, which had a mean final abundance of 1,844 males, and 26.3% of the simulations fell below 1,000 males. Population vulnerability increased when additive models contained the lower 25th and 10th percentiles of projected end‐of‐century changes in March precipitation. For example, when the lower 10th percentile of projected changes in precipitation was added to a reduction in breeding grounds habitat quantity due to climate change, 98.1% of simulations fell below 200 males, and 9.9% went extinct (Table 2).

## 4 DISCUSSION

Our study indicates the Kirtland's Warbler population is stable under current environmental and management conditions, consistent with our previous simulation study (Brown et al., 2017) and long‐term monitoring data (U.S. Fish & Wildlife Service, 2015). Based on our results, probability of long‐term persistence for this species will depend on how wintering grounds habitat quality changes (i.e., how much March precipitation changes in the central Bahamas). Quantity of breeding grounds habitat was an important determinant of whether or not the population remained above the management target of 1,000 breeding males, but loss of breeding habitat alone (up to 48.7%) did not cause extinction.

This study represents one of the first attempts to integrate full annual cycle dynamics into a migratory bird population simulation model (Hostetler, Sillett, & Marra, 2015). Many studies have indicated that accounting for environmental conditions on the wintering grounds and during migratory periods is important for understanding population dynamics of migratory birds (e.g., Balbontin et al., 2009; Drake, Rock, Quinlan, Martin, & Green, 2014; Sheehy, Taylor, & Norris, 2011). For example, an analysis of long‐term monitoring data for the Canada Warbler (*Cardellina canadensis*) indicated that declining trends were associated with reduced survival during the nonbreeding period (Wilson et al., 2018), and analyses of long‐term monitoring data for the American Redstart (*Setophaga ruticilla*) showed that temporal variation in wintering grounds habitat quality was a strong predictor of changes in abundance on the breeding grounds (Wilson, LaDeau, Tottrup, & Marra, 2011). Kirtland's Warbler is one of the few species with quantitative empirical models that link environmental conditions on the wintering grounds to population vital rates (Rockwell et al., 2012, 2017). Given the apparent high importance of wintering grounds habitat quality to long‐term population viability of Kirtland's Warbler and other migratory bird species of conservation concern (e.g., Wood Thrush [*Hylocichla mustelina*]; Rushing, Ryder, & Marra, 2016; Taylor & Stutchbury, 2016), we encourage researchers studying migratory songbirds to focus efforts on quantifying effects of environmental conditions outside of the breeding season on population vital rates.

While our model only considered the impacts of decreased precipitation on the wintering grounds, temperature is also projected to increase (Wolcott et al., 2018). Even if mean March precipitation remains constant, arthropod and fruit availability will likely decrease during this critical premigration period due to increased temperature stress and evapotranspiration rates (Bounoua et al., 1999; Lister & Garcia, 2018). Thus, while the median projected end‐of‐century change in March precipitation did not indicate the population was at risk of extinction, this should be considered a conservative projection. Additional research is needed to quantify how temperature and precipitation interact to influence food abundance for Kirtland's Warbler (Wunderle et al., 2014).

The potential future scenarios we modeled in this study were based on the assumption that managers would not be responsive to reductions in breeding grounds habitat quantity caused by a contraction in the suitable climate zone for jack pine or unsuitability of experimental plantations. Realistically, we would expect managers to respond by changing the spatial distribution of KWMAs or discontinuing unsuitable experimental plantation designs, respectively. The purpose of these scenarios was to quantify the importance of breeding habitat quantity for maintaining a robust Kirtland's Warbler population. For this study, we also assumed that Kirtland's Warblers will not adapt to climate changes by modifying their wintering distribution, breeding distribution, or temporal activity cycle. We acknowledge that long‐term bird monitoring data sets indicate distributional shifts in response to climate change are already occurring for some species (e.g., La Sorte & Thompson, 2007; Zuckerberg, Woods, & Porter, 2009). However, thus far there is no indication that the wintering distribution of Kirtland's Warblers is changing, and without additional human intervention, there is little opportunity for a major distributional change on the breeding grounds due to strict habitat requirements. Further, the LP of Michigan will likely continue be the most suitable region for jack pine in the Upper Midwest, USA (Donner et al., 2018).

In conclusion, our study indicates the Kirtland's Warbler is stable under current environmental and management conditions, is vulnerable to climate changes on the wintering grounds, and will continue to be reliant on humans to maintain the current quantity of breeding habitat to minimize the risk of the population falling below specified management thresholds. This study represents the final component of a Kirtland's Warbler research initiative that also included assessing vulnerability to management changes under contemporary climate conditions (Brown et al., 2017), projecting impacts of climate change on breeding grounds habitat (Donner et al., 2018), and projecting impacts of climate change on wintering grounds habitat (Wolcott et al., 2018).

## CONFLICT OF INTEREST

The authors declare they have no conflicting interests with the work herein.

## AUTHOR CONTRIBUTIONS

DJB, DMD, CAR, and CIB conceived and designed the study. DJB constructed the model, performed simulations, and drafted the manuscript. DMD, CAR, and CIB edited the manuscript. All authors read and approved the final product.

## Figures and Tables

**Figure A1:**
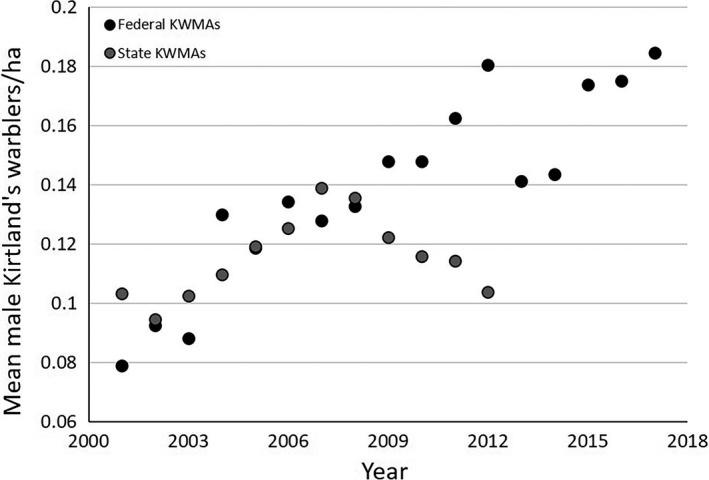
Mean density of male Kirtland's Warblers (*Setophaga kirtlandii*) on state and federal Kirtland's Warbler Management Areas (KWMA) in the northern Lower Peninsula of Michigan, USA, between 2001 and 2017. We derived the 2001–2012 data from Huber et al. (2013). Data from 2013 to 2017 are used with permission from Philip Huber and Kimberly Piccolo, U.S. Forest Service, Huron‐Manistee National Forest

## Data Availability

STELLA code archived in the Dryad Digital Repository, https://doi.org/10.5061/dryad.1536b56.

## Appendix 1

### ANNUAL PRODUCTIVITY ESTIMATION

Kirtland's Warbler (*Setophaga kirtlandii*) is a breeding grounds habitat specialist, restricted to large stands of young, dense jack pine (*Pinus banksiana*) in glacial outwash plains containing well‐drained sandy soils (Probst, 1988; Probst et al., 2003). Ecologically, young jack pine trees provide low hanging branches that Kirtland's Warblers use for concealment of their ground nests, and well‐drained sandy soils reduce the probability that nests will become inundated with water during heavy rainfall events (Mayfield, 1960; Probst, 1988). Males establish breeding territories in jack pine‐dominated patches that are typically larger than 32 ha, with tree densities greater than 2000 stems per ha, and trees ca. 5–23 years old (Probst, 1988; Probst & Weinrich, 1993; Walkinshaw, 1983). Dense jack pine stands were historically generated naturally through large wildfires with ca. 60 year return intervals (Cleland et al., 2004; Mayfield, 1993), but due to broad‐scale fire suppression, an extensive network of jack pine plantations is currently used to maintain large amounts of suitable habitat on the landscape (Donner et al., 2009; Probst & Weinrich, 1993).

To estimate annual variation in quality of breeding habitat, we identified suitable jack pine stands using habitat management program records that included stand year‐of‐origin and habitat regeneration‐type (i.e., clear‐cut and natural regeneration, plantation, wildfire). The habitat regeneration types represent a gradient of tree densities, with natural regeneration stands typically having low tree densities (<2,200 trees/ha), and plantation/wildfire stands typically having high tree densities (2,500 to >7,500 trees/ha; Probst & Weinrich, 1993). Previous studies using stand history data defined Kirtland's Warbler habitat suitability based on stand age (Donner, Ribic, & Probst, 2009, 2010). However, jack pine growth rates are known to differ latitudinally and longitudinally due to variations in temperature, precipitation, and soil types (e.g., Kashian, Barnes, & Walker, 2003). Thus, we translated known stand ages across the study area to estimated stand heights to more accurately capture patch‐specific habitat suitability over time. The stands were classified as nonhabitat (0), low (1)‐, moderate (2)‐, or high (3)‐quality habitat each year based on regeneration‐type and estimated stand height (see [Brown et al., 2017] for additional details).

To allow both breeding grounds habitat suitability and wintering grounds precipitation to predict productivity, our equation included two predictive components. The first component estimated baseline productivity based on breeding grounds habitat quality. The intercept of this equation (i.e., 1.1114) represented the baseline number of male fledglings produced per male each year. We then multiplied the intercept by the pairing success estimates for low/moderate suitability and high suitability breeding habitat, modified to account for polygyny in high‐quality habitat (Bocetti, 1994; Probst & Hayes, 1987; Rockwell, 2013). We used 0.85 for habitat suitability values ≤2 based on the estimate of Probst (1986). We used 0.97 for habitat suitability value = 3 based on Rockwell (2013). This resulted in baseline productivity values of 0.9447 and 1.0781 for birds occupying low/moderate‐quality and high‐quality habitat, respectively. We used a regression equation to estimate baseline productivity values from mean habitat quality: Baseline productivity = 0.678 + (0.1334 × [Habitat Quality]). We did not model nest failure as a function of habitat quality because nest parasitism rates are currently < 1% (Rockwell, 2013), and probability of nest loss does not appear to differ based on habitat quality or stand age (Bocetti, 1994).

For the second component of the equation, we used the slope of the wintering grounds precipitation–productivity equation to estimate the number of new males produced above the baseline per cm of precipitation: Additional productivity = 0.168 x [precipitation (cm)]. Combining the influence of breeding and wintering grounds habitat quality on annual productivity: Productivity = (0.678 + (0.1334 × [Habitat Quality])) + (0.168 × [precipitation (cm)]). Because there is no upper bound to this equation, we bounded maximum fledgling production by the largest empirical estimate (i.e., 2.19 male fledglings per male; Shake & Mattsson, 1975). To simulate baseline annual habitat quality, we used a Gaussian distribution parameterized using the estimated mean habitat quality across all suitable jack pine stands in the northern Lower Peninsula of Michigan from 2004 to 2013 (mean = 2.35, *SD* = 0.05).

### BREEDING GROUNDS CARRYING CAPACITY ESTIMATION

Annual breeding grounds carrying capacity is influenced by quantity and quality of breeding habitat, with quality reflected by density of Kirtland's Warblers (Bocetti, 1994; Probst, 1986). Huber, Kintigh, and Sjogren (2013) quantified mean density of male Kirtland's Warblers across all occupied stands on state and federal Kirtland's Warbler Management Areas (KWMA) in the northern Lower Peninsula of Michigan, USA, between 2001 and 2012, and the U.S. Forest Service continued to quantify mean density on federal KWMAs through 2017. These data show that annual mean density is typically higher on federal KWMAs (located in the core of the breeding range) and that mean density on federal KWMAs increased by 32% between 2003 and 2004 (Figure A1). For this study, we characterized annual variation in density using annual density estimates from 2004 to 2017. To estimate annual density for state land from 2013 to 2017, we used the mean of the observed densities on state land from 2004 to 2012. We then computed average annual density for the northern Lower Peninsula (LP) of Michigan as the mean of the state and federal land estimates, weighted by proportion of occupied habitat on state and federal land. The mean density was 0.1307 ± 0.0076 males/ha.

The Kirtland's Warbler Conservation Team defined 14,593 ha as the target for annual breeding habitat availability (U.S. Fish & Wildlife Service, 2018), and we used this as our baseline annual breeding habitat quantity. To obtain annual carrying capacity, we multiplied quantity of breeding habitat by estimated density: 1,907 ± 111 males. Thus, the carrying capacity model accounted for annual variability in mean quality of habitat, reflected in changes in mean density. When the model population exceeded the carrying capacity, reproduction was restricted to the carrying capacity, with the remaining individuals allowed to survive, but not reproduce.

### WINTERING GROUNDS CLIMATE CHANGE PROJECTIONS

To estimate potential climate change impacts on wintering grounds habitat quality, we projected proportional changes in average March precipitation in 2100 relative to baseline precipitation for the focal Bahamian islands. The spatial data used were derived from the most current General Circulation Models (GCM) developed for the fifth Coupled Model Inter‐comparison Project (CMIP5), which corresponds to Assessment Report 5 of the IPCC (IPCC, 2013). These GCM data were statistically downscaled from 0.5° × 0.5° (ca. 50 km × 50 km) resolution to 0.008° × 0.008° (ca. 1 km × 1 km) using pattern scaling (Tebaldi & Arblaster, 2014). The baseline precipitation and temperature data were derived from the 20‐year observation period between 1986 and 2005 (IPCC, 2013).

We used an ensemble of 40 GCMs, with mid‐equilibrium climate sensitivity levels (equilibrium climate sensitivity represents the expected atmospheric warming in response to a doubling of CO_2_ [IPCC, 2013]), and Representative Concentration Pathway (RCP) 8.5, which assumes CO_2_ emissions will continue to increase over the next century (Moss et al., 2010). We used the median (−0.055), lower 25th percentile (−0.331), and lower 10th percentile (−0.558) of projected end‐of‐century changes in March precipitation based on the GCM ensemble. Additional information on the climate change projections used here can be found in Wolcott et al. (2018). We note there is much greater uncertainty for projecting future precipitation compared to temperature, and some models did project increases in March precipitation. However, we did not include scenarios where precipitation increases because the Kirtland's Warbler population is already stable with current wintering grounds precipitation levels (Brown et al., 2017).
